# Interaction Between a Bacterivorous Ciliate *Aspidisca cicada* and a Rotifer *Lecane inermis*: Doozers and Fraggles in Aquatic Flocs

**DOI:** 10.1007/s00248-017-1036-5

**Published:** 2017-07-19

**Authors:** Aleksandra Walczyńska, Mateusz Sobczyk, Edyta Fiałkowska, Agnieszka Pajdak-Stós, Janusz Fyda, Krzysztof Wiąckowski

**Affiliations:** Institute of Environmental Sciences, Jagiellonian University, Gronostajowa 7, 30-387 Kraków, Poland

**Keywords:** Activated sludge, *Aspidisca*, Ciliates, Flocs, *Lecane inermis*, Macroaggregates, Scavenging, Wastewater treatment

## Abstract

Activated sludge is a semi-natural habitat composed of macroaggregates made by flocculating bacteria and inhabited by numerous protozoans and metazoans, creating a complicated interactome. The activated sludge resembles the biological formation of naturally occurring floc habitats, such as “marine snow.” So far, these two types of habitat have been analyzed separately, despite their similarities. We examined the effect of a bacterivorous ciliate, *Aspidisca cicada*, on the quality of the macroaggregate ecosystem by estimating (i) the floc characteristics, (ii) the proliferation of other bacterivores (rotifers), and (iii) the chemical processes. We found that *A. cicada* (i) positively affected floc quality by creating flocs of larger size; (ii) promoted the population growth of the rotifer *Lecane inermis*, an important biological agent in activated sludge systems; and (iii) increased the efficiency of ammonia removal while at the same time improving the oxygen conditions. The effect of *A. cicada* was detectable long after its disappearance from the system. We therefore claim that *A. cicada* is a very specialized scavenger of flocs with a key role in floc ecosystem functioning. These results may be relevant to the ecology of any natural and engineered aggregates.

## Introduction

Suspended organic particles and aggregate-associated microbial processes are of fundamental importance in any aquatic ecosystem. Because of their resemblance to snowflakes, larger aggregates in the water column have been called “marine snow” [1, 2], “lake snow” [3], or “river snow” [4, 5]. Particles that form such “aquatic snow” are of various origins depending on the system and season [6]. Although organic aggregates in water may be formed by a variety of physicochemical processes, the activity of microorganisms, in particular bacteria and algae, seems to be of particular importance to their characteristics and fate [7]. Depending on the origin and quality of these particles, the sedimenting “snow” flocs can release or adsorb labile organics and mineral substances from the surrounding water [3, 8, 9]. Much of the transport of the organic carbon and nutrients from the surface to the deep layers of oceans is due to the sedimentation of “snow” particles [6, 9, 10].

Flocculation enhanced by microbial activity is also the basic mechanism in activated sludge, the most popular wastewater treatment technology. The activated sludge has been perceived as a dynamic process in wastewater treatment since the experiments conducted by Arden and Lockett [11], [after 12] with two main functions: the biodegradation of soluble organic matter through oxidation and the separation of the newly formed biomass through flocculation and sedimentation [13]. Currently, activated sludge is instead considered to be a specific, semi-natural habitat where biological processes of micro-organismal flocculation are supported by human interference through mixing and aerating to create an effective surface for the adsorption of dissolved and colloidal particles [14]. Shifting the approach from the perspective of the process (changes in time) to a state (dynamics in space) enables an understanding of the complex interactions involved and, further, the identification of weak and strong links between the biological compartments.

Suspended flocculated aggregates are hotspots of microbial activity [1, 6, 7, 15]. This activity is associated with the production of exopolymers (EPS), the substances promoting the aggregation of organic matter particles [16]. Large numbers of bacteria and bacterial aggregates attract numerous bacterivores and predators [7, 17, 18]. Both natural and engineered flocculated aggregates are usually colonized by numerous heterotrophic flagellates, amoebae, ciliates, and small metazoans. The selective grazing by protozoans on bacteria per se and on the EPS the bacteria produce affects the floc size, shape, and, in consequence, its functional characteristics. The most important role is played by ciliates, termed the “engineers of biofilms” [19] due to their properties in increasing the habitat heterogeneity in macroaggregate systems [20, 21]. Liss et al. [22] studied the ultrastructure of the flocs from engineered and natural (riverine) systems. They concluded that both floc types when viewed at high resolution (1 nm) resembled microbial biofilms; hence, they proposed analogy that “flocs might be envisioned as biofilms turned back on themselves so that the biofilm/substratum interface is internalized as the core of a suspended floc”. The more compact, robust, and regular the flocs, the better the quality of the activated sludge [23, 24]. Because the size and shape of flocs generally affect their adsorptive properties and settleability, these factors also influence the transport of substances from the water surface to deeper water layers and are therefore important floc features in any given habitat.

Although research on activated sludge has been carried out for a long time and the significant effect of protozoans on the treatment process has been acknowledged, the actual mechanisms behind this process remain largely unknown. For example, we do not know to what extent the presence of a particular species or a functional group can affect the biological compartment of activated sludge. The great diversity of component organisms suggests a plethora of possible relationships within the food chain [25], with complicated associations between bacteria and bacterivores [19, 26, 27], within bacterivores [28–31], and between bacterivores and their predators [32]. However, similar to all ecological systems, one might expect that not all of these organisms are of equal importance. Identifying the species and interactions that are responsible for the observed structure and function is essential to understanding any floc habitat.

Our own experience and several literature references point to the crawling ciliates *Aspidisca* sp. as being potentially important agents in the activated sludge community. Species of *Aspidisca* are particularly common and are often among the most abundant ciliates in various activated sludge systems [33–35]. Interestingly, various *Aspidisca* species have also been observed on marine snow particles, indicating a possible affinity to this particular type of habitat [17, 36]. The relatively small body size of *Aspidisca* sp. in comparison to other sludge bacterivores [37] and its obligatory affinity for floc bacteria [38] suggest the possibility of a specific ecological function of this genus. *Aspidisca* sp. appears to be the most effective in flocculation and in substrate removal when compared to four other ciliate species [38]. It has also been related to efficient ammonia removal, as is shown in a study on nitrifier succession in a newly opened wastewater treatment plant (WWTP) [26], and was the most effective in nutrient removal in comparison to two other protozoans [39].

The aim of the present study was to estimate the effect of *Aspidisca cicada* on the macroaggregate system. This effect was measured under laboratory conditions with three different traits investigated as being affected by the presence of *A. cicada*: (1) floc morphology, (2) the proliferation of *Lecane inermis* rotifers, and (3) the chemical properties of the supernatant. Both floc characteristics and the chemistry of the supernatant remaining after sludge sedimentation are widely used for the assessment of the quality of activated sludge systems [23, 24]. The proliferation of the rotifer *L. inermis* was introduced as another system quality trait because this species has been previously shown to significantly affect the properties of activated sludge by eliminating filamentous organisms [27]. Using such a comprehensive evaluation, we intended to reveal whether *A. cicada*, with its unique characteristics among ciliates, positively affects the floc ecosystem by improving the conditions for other bacterivore species (*L. inermis*) through floc engineering (floc characteristics) and by selecting specific bacteria (chemical properties as an indirect measure of bacterial processes). The results of this study have universal ecological implications for semi-natural (activated sludge) types of floc systems, but may also be applicable to natural aggregates such as aquatic snow particles. To the best of our knowledge, we made the first attempt to describe a robust biological relationship from among the plethora of sensitive, complicated relationships within the biological compartment of any type of floc ecosystems.

## Methods

### Study Outline

#### Study Species

*A. cicada* is a small crawling ciliate with a body length of 25–40 μm and a mouth size of 3 × 5 μm [40]. *Aspidisca* ciliates are believed to collect their food particles from surfaces using a peculiarly reduced adoral zone of membranelles [41]. Three *Aspidisca* species have been found in activated sludge systems: *A. cicada*, *A. lynceus*, and *A. turrita*, but the first occurs more frequently in activated sludge while the two others seem to be rarer [42]. *Aspidisca* sp. is stenophagic in terms of the types of bacteria used as a food source [43]. We isolated *A. cicada* individuals from activated sludge samples that originated from a treatment plant in southern Poland. The ciliates were cultured according to Sudo and Aiba [43] with some modifications. We kept *A. cicada* in small glass petri dishes (*ø* = 60 mm) with three to five cover slips (20 × 20 mm) on the bottom, at room temperature. The cover slips were used as “inoculation glasses” in our experiments. The ciliates in the laboratory culture were initially fed with the bacterium *Enterobacter* sp. The method of *A. cicada* culturing changed between experiments 1 and 2 so that they were later fed with a mixture of bacteria isolated from the treatment plant of ciliate origin. These bacteria were isolated and cultivated according to Sudo and Aiba [43]. Lecane inermis is a bacterivorous, eurioic, monogonont rotifer that lives in psammon in fresh and salt water reservoirs [44] and also frequently and abundantly occurs in activated sludge system. This species was found to effectively feed on filamentous bacteria [27], whose blooms are the main cause of the troublesome effect of “sludge bulking” worldwide [23, 24]. The ability to feed on filamentous bacteria is relatively rare in activated sludge [27 and citations therein], and therefore, *L. inermis* acts as an important biological agent in such systems. The clone of the rotifers used for the experiment was isolated from a WWTP in southern Poland and was thereafter cultured in petri dishes filled with spring water (Żywiec brand, Poland; see Appendix Table 3 for the information on mineral contents), fed with a nutritional powder (patent procedure pending EPO EP 14731401.7) and kept in darkness at 20 °C.

#### Experimental Setup

In order to identify the effect of *A. cicada* on the three aspects of the quality of the macroaggregate system, *A. cicada* was cultured alone or together with *L. inermis* in Erlenmeyer flasks in 150 ml of Żywiec spring water as a medium and a nutritional powder suspension as a food source, at room temperature (approximately 21 °C) and under natural photoperiod. The clonal laboratory cultures of both species were used. Throughout the experiments, the flasks were shaken on a GFL 3017 laboratory shaker (110 rpm). Three experiments were conducted, each 3 weeks long:

Experiment 1: Four treatments of batch culture (three replicates each) were established: control (no *L. inermis* or *A. cicada*; C), the presence of *A. cicada* (A), the presence of *L. inermis* (L), and the presence of both *A. cicada* and *L. inermis* (AL). The initial number of *L. inermis* in treatments L and AL was 50 individuals per ml (hereafter, ind./ml). We added two inoculation glasses with *A. cicada* (approximately 10 ind./ml of the initial concentration) to the A and AL treatments. The experiment was continued until *A. cicada* and *L. inermis* disappeared from the system in AL treatment. During the experiment, subsamples were taken for floc structure and rotifer growth analyses (details are described in the next section) every 3 to 4 days. After each sampling event, 3 ml of supernatant was removed and replaced with 3 ml of our standard food suspension (1 × basic concentration) inoculated with *Enterobacter* sp.

Experiment 2: The same treatments and conditions were maintained as those in the previous experiment except for two changes: (i) we replaced the batch cultures with semi-continuous cultures by removing supernatant in the amount of one third of the flask volume (50 ml) after each sampling event and replacing it with 47 ml of fresh medium and 3 ml of fresh food suspension and (ii) we removed the possible effect of a shortage of food by adding food proportionally to the increasing rotifer number from 1× to 5× the basic concentration each time dissolved in the same volume of 3 ml and inoculated with *Enterobacter* sp. Sampling every 3 to 4 days was carried out until *L. inermis* established a stable population.

Experiment 3: The effect of different numbers of *A. cicada* on *L. inermis* population growth in semi-continuous cultures was examined. For this, one third of the supernatant was removed at each sampling event and replaced with fresh medium. In four Erlenmeyer flasks, the *A. cicada* cultures were started from a different number of inoculation glasses (0, 2, 4, or 6) with 147 ml of spring water medium and 3 ml of basic food concentration. After 2 days of *A. cicada* proliferation, the same number of cultured *L. inermis* (50 ind./ml) was added to each flask. We estimated the number of rotifers every day until the number of *A. cicada* reached its peak and then every 3–4 days until *A. cicada* disappeared from the experimental flasks.

### Measurement of the Effects of *Aspidisca cicada*

#### Floc Structure Analysis

At each sampling event during experiments 1 and 2, 0.5 ml of the thoroughly shaken cultures were taken from each treatment. The subsamples were fixed with Lugol solution (25 μl per well) and images of all of samples were taken with a magnification of 6.5× using a stereomicroscope (Zeiss Stemi 2000-C, Carl Zeiss AG, Germany) and camera setup (PixeLink, PixeLINK®, Canada), using the software NIS-Elements (Nikon, Japan). In experiment 1, the photos were taken directly from the plate wells, in a way enabling the coverage of a whole well. In experiment 2, each sample was removed from a well and placed on a slide glass, while the camera was adjusted to take photos of whole drops. The assessment of floc attributes was based on two traits: size and compactness. Each trait was assessed on a 0–5 scale using a modified method of a standard procedure used in activated sludge analyses [23]. In this method, increasing values denote a larger size and higher level of compactness. To make the estimates more objective, the coded floc images were presented to two persons skilled in floc structure assessment who had no previous knowledge of the treatments applied.

To make the floc size comparable with other studies, it was determined by the longest diameter using the NIS-Elements software and was measured only for experiment 2 for all measurable flocs from each replicate on the sampling date at which the peak in *A. cicada* numbers occurred. Two traits characterizing floc structure, size, and compactness were averaged for two estimates and analyzed separately using repeated measures ANOVA (PROC GLM, SAS v. 9.4, SAS Institute, Cary, NC, USA), with treatment as a between-subject factor and sampling date as a within-subject factor. In cases in which the sphericity assumption, important for repeated measures ANOVA, was not met, the *p* values were corrected using a Huynh-Feldt-Lecoutre epsilon correction [45].

#### Rotifer Growth

At each sampling event during all three experiments, four subsamples of 25 μl each (100 μl in total per replicate) were taken to count individuals [standard method; 46]. The subsamples were fixed with Lugol solution and all rotifers were counted on glass slides under an inverted microscope (IMT2 Olympus, Japan and IX 71 Olympus, Japan). The change in rotifer numbers over time was statistically analyzed using repeated measures ANOVA (PROC GLM, SAS).

#### Chemical Parameters

Chemical oxygen demand (COD) is used to measure the amount of dissolved organic matter (DOM) in water (oxygen needed to reduce organic matter in water by chemical methods) and is a useful measure of wastewater quality [47]. COD was used to estimate the DOM concentration in the supernatant (i.e., sludge activity). Three parameters, nitrate and ammonium concentration, which provide information on nitrification processes, as well as COD were estimated on the last day of experiment 1 and at the peak of *A. cicada* numbers in experiment 2. Additionally, total phosphorus was analyzed in experiment 2. The 50 ml subsamples for chemical analysis were taken from medium after 30 min of sedimentation. All chemical compounds were analyzed according to standard methods using spectrophotometry [48]. Each parameter was analyzed with the Kruskal-Wallis test to identify statistical differences among the treatments (Statistica 64, v. 10; StatSoft).

## Results

### Population Growth Rates

**Fig. 1:**
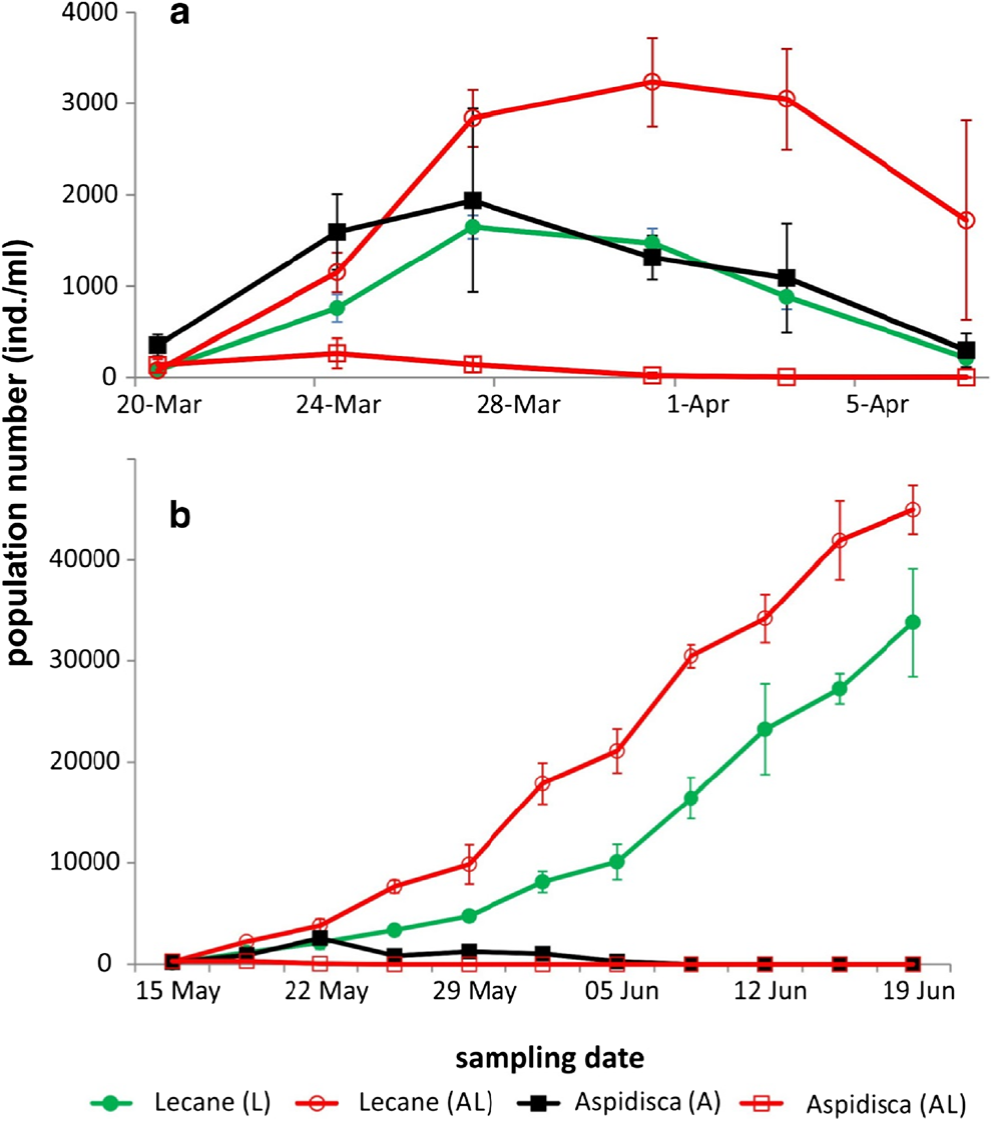
The population numbers of *Aspidisca* and *L. inermis* in experiment 1 (**a**) and experiment 2 (**b**). Mean ± SD. A: *Aspidisca cicada* monoculture, L: *Lecane inermis* monoculture, AL: *A. cicada* and *L. inermis* mixed culture

During all three experiments, the added *A. cicada* populations crashed, but this occurred earlier when cultured together with *L. inermis* compared to when cultured alone (Fig. 1). The maximal number of *A. cicada* in treatment A was sevenfold (experiment 1) or eightfold (experiment 2) larger compared to the number in treatment AL.

**Fig. 2:**
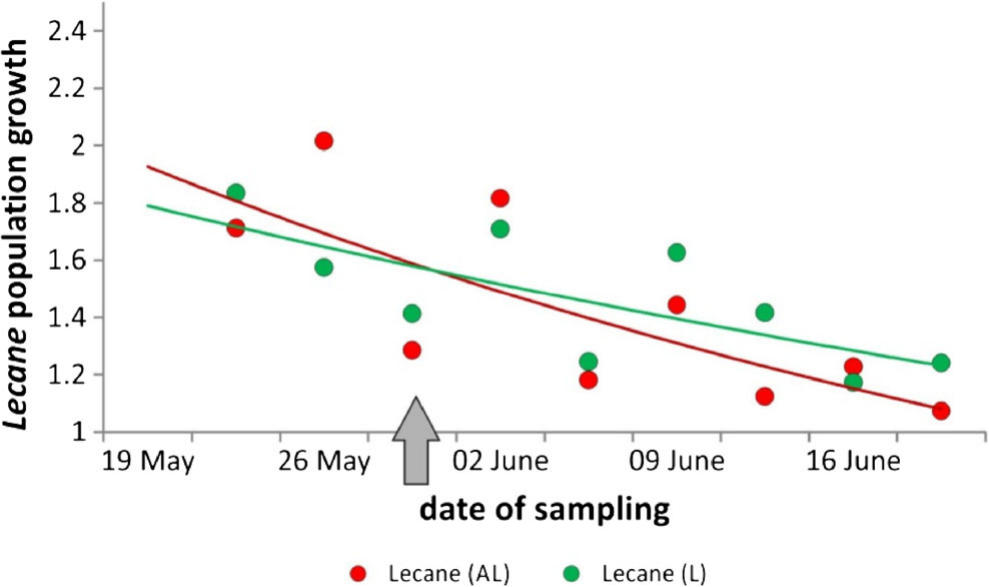
The exponential estimation of the population growth (the number at time *T*_*x*+1_ divided by the number at time *T*_*x*_) of the rotifer *L. inermis* in experiment 2. The *gray arrow* denotes the moment *Aspidisca cicada* disappears from the treatment AL. L: *L. inermis* monoculture, AL: *Aspidisca* and *L. inermis* mixed culture

In the batch cultures in experiment 1, *L. inermis* achieved a twofold higher number in AL than in the L treatment and then decreased in number in both treatments. In the semi-continuous cultures in experiment 2, *L. inermis* increased continuously in number in both AL and L treatments, reaching approximately 45,000 ind./ml at the end of the experiment (Fig. 1). The rate of rotifer population growth was higher in the AL treatment until *A. cicada* disappeared from the system, and only after that did the rotifers in the L treatment start to prevail in growth rate, as calculated by subtracting counts from consecutive sampling events (Fig. 2). In experiment 3, in which rotifers were added to treatments A0, A2, A4, and A6 when *A. cicada* density was 0, 80, 170, and 230 ind./ml, respectively, *A. cicada* reached its maximal number on the sixth or seventh day depending on the treatment. The *L. inermis* population continued exponentially increasing for the following 2.5 weeks (data not shown).

In experiment 1, considerable contamination by ciliates of the *Cyclidium* genus was detected in treatments L and AL, while contamination by crawling ciliates of the genus *Chilodonella* and attached ciliates was observed in treatments A and AL in experiments 1 and 2. Yet, we claim that neither *Cyclidium* nor *Chilodonella* could have considerable effect on flocculation; *Cyclidium* is a swimming ciliate occupying different feeding niche, not forming flocs [49], while *Chilodonella*, a crawling ciliate that could potentially compete with *Aspidisca*, occurred in smaller numbers than *Aspidisca*, especially in treatment A. Other protists present in the experimental cultures were small (<20 μm) heterotrophic flagellates (not detected in treatment A) and large (>20 μm) flagellates in experiment 1, treatment L. The control cultures were occasionally contaminated with naked and testate amoebae.

### Floc Structure Analysis

The characteristics of flocs differed considerably among the treatments (Appendix Table 4). The mean floc diameters (mean ± SD) estimated for all treatments in experiment 2 from the smallest to the largest were 318 μm ± 66 (L), 422 μm ± 150 (C), 514 μm ± 126 (AL), and 723 μm ± 210 (A). These flocs are categorized as large according to the guidelines of Eikelboom [23] or medium (L and C) and large (A and AL) according to Jenkins et al. [24].

**Fig. 3:**
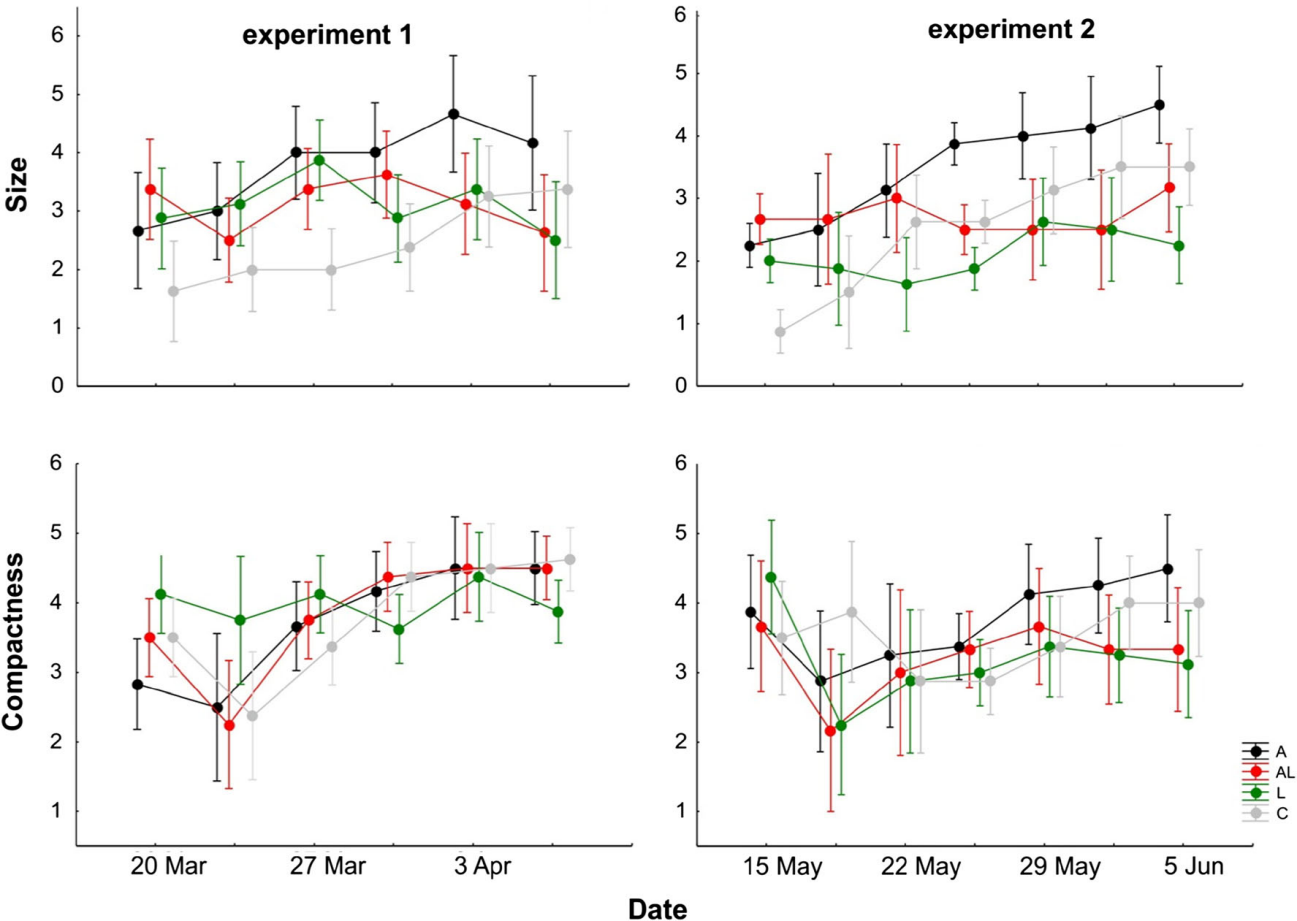
The changes over time for two floc characteristics, size (*upper row*) and compactness (*lower row*), both estimated on a 0–5 scale. Means ± 0.95 confidence interval (CI). A: *Aspidisca cicada* monoculture, L: *Lecane inermis* monoculture, AL: *A. cicada* and *L. inermis* mixed culture, C: Control treatment

The results of the RM-ANOVAs for the qualitative floc traits were very similar for experiments 1 and 2 (Table 1). Floc size differed significantly across the treatments and was affected differently by time for each treatment (significant time × treatment interaction). Floc compactness did not differ among the treatments but was affected by time, with experiment-dependent significance of the time × treatment interaction (Table 1). Excluding the control treatment from the analyses did not change the results. The flocs constructed by *A. cicada* (A) were the largest in both experiments, while AL flocs were similar (experiment 1) or larger (experiment 2) in size than L flocs (Fig. 3). Regarding compactness, L flocs tended to be the least compact in both experiments, especially in their later stages, while A flocs tended to be similar (experiment 1) or denser (experiment 2) than AL flocs (Fig. 3). There is a general increasing trend in compactness over time for all treatments except for L flocs in experiment 1. Neither the size nor compactness of flocs can be statistically compared between the experiments because different methods for capturing images were used (see the “Methods”).

**Table 1:** The results of the repeated measures ANOVA for floc size and floc compactness in experiments 1 and 2

Analyzed trait	Factor	Experiment 1	Experiment 2
		*p* value	
Floc size	Treatment	*0.0033*	<*0.001*
	Time	*0.0031*	<*0.001*
	Treatment × time	*0.0141*	*0.0021*
Floc compactness	Treatment	0.5752	0.0871
	Time	<*0.001*	*0.0007*
	Treatment × time	*0.0045*	0.1824

Significant effects are italized

The occasional contaminants in treatment C caused considerable variation among replicates (Appendix Table 4). The flocculating agents in this treatment, in addition to bacteria, were mostly naked amoebae, small testate amoebae, and flagellates and simply the process of shaking. The size of C flocs was the smallest at the beginning and increased over time but never reached the size of the flocs made by *A. cicada* alone (both experiments; Fig. 3). The compactness of C flocs was comparable to that of A and AL flocs in experiment 1 and was lower than that of A flocs in experiment 2 (Fig. 3).

### Rotifer Growth

The rotifers proliferated better in the AL treatment compared to the L treatment in both experiments (Fig. 1), but only the data from experiment 2, where rotifers did not vanish from the cultures, were statistically analyzed. The population growth of rotifers, analyzed till the date of *Aspidisca* disappearance from the system (5 June), was significantly faster in treatment AL than in treatment L (*F*_(1,6)_ = 83.58; *p* < 0.001). This pattern was affected by time (*F*_(6,36)_ = 286.63; *p* < 0.001) and its interaction with treatment (*F*_(6,36)_ = 38.81; *p* < 0.001).

**Fig. 4:**
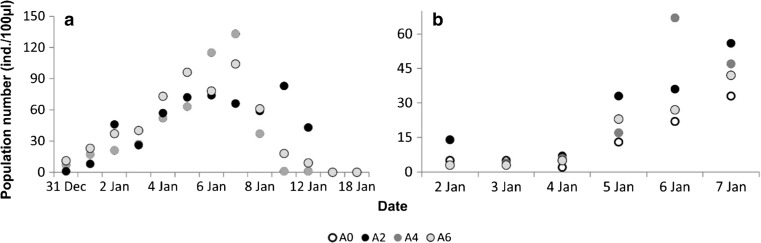
The population numbers of *Aspidisca* (**a**) and *L. inermis* (**b**) in experiment 3. The dynamics of *L. inermis* population growth are shown for the period until the *A. cicada* population reached its maximal number. A0, A2, A4, and A6 denote the treatments, which represent the initial number of inoculation glasses with *Aspidisca* culture of 0, 2, 4, and 6, respectively

In experiment 3, when analyzing the initial phase until *A. cicada* reached its maximal number (the seventh day from the onset of experiment), rotifer growth was the slowest in the absence of *A. cicada* (A0), intermediate for mid- and high initial numbers of *A. cicada* (A4 and A6), and the fastest in the treatment in which the initial number of *A. cicada* was the lowest (A2; Fig. 4).

### Chemical Parameters

**Fig. 5:**
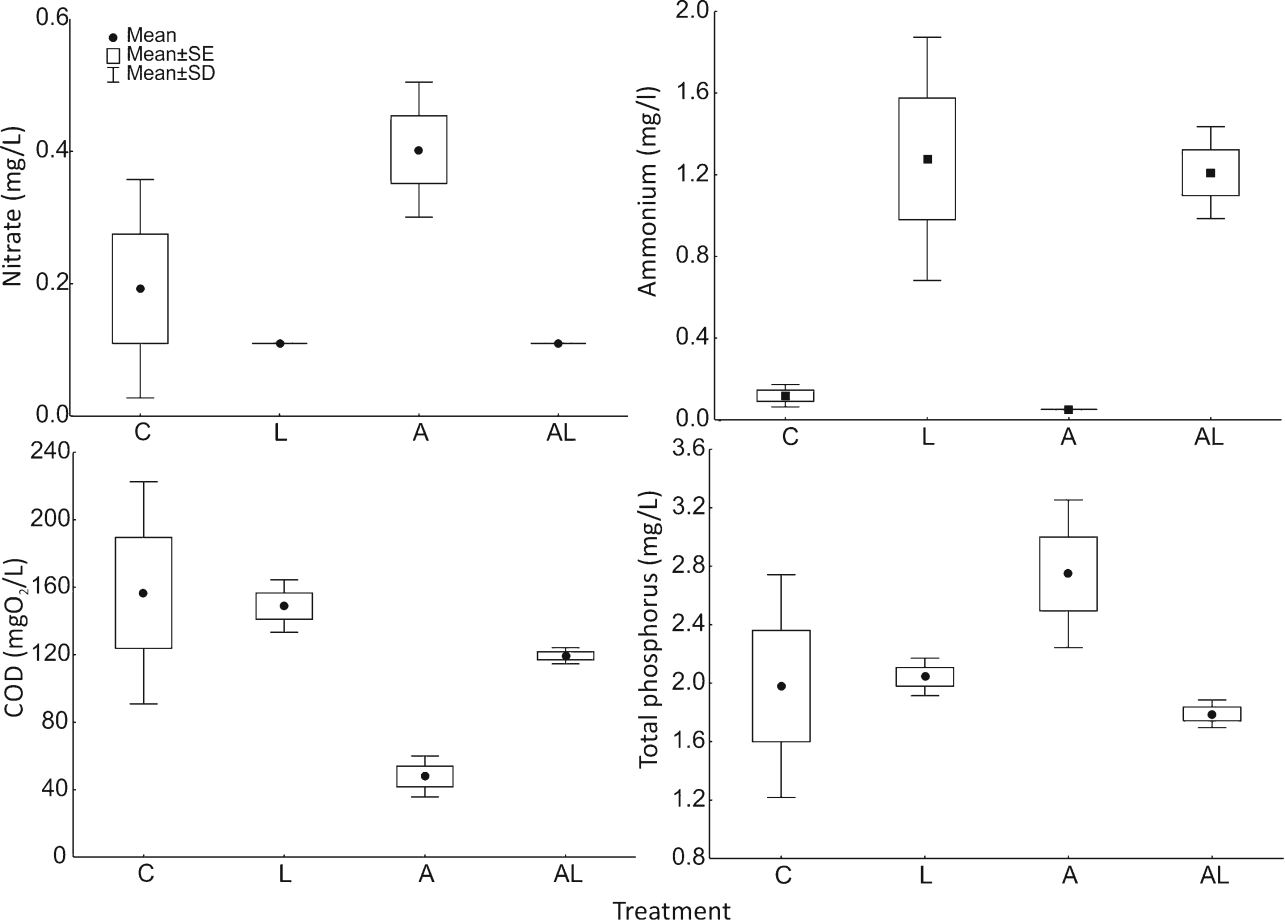
The chemical parameters estimated for experiment 2: nitrate, ammonium, and total phosphorus concentrations, as well as chemical oxygen demand (COD). A: *Aspidisca cicada* monoculture, L: *Lecane inermis* monoculture, AL: *A. cicada and L. inermis* mixed culture, C: control treatment

The qualitative pattern of the differences among the treatments was consistent for both experiments, with the exception of nitrate (Table 2). Nitrate did not differ significantly in experiment 1 but differed in experiment 2. Because all of the other results were qualitatively similar, only the results for experiment 2 are presented. Nitrate concentration differed among the treatments (*H*_(3,16)_ = 10.86; *p* = 0.0125), with the highest value achieved in the A treatment and comparably low values in the L and AL treatments (Fig. 5). Ammonium concentration differed among the treatments (*H*_(3,16)_ = 12.92; *p* = 0.0048) and was the lowest in the A treatment with comparably high values in the L and AL treatments (Fig. 5). COD differed among the treatments (*H*_(3,16)_ = 10.94; *p* = 0.0120) and was the lowest in the A treatment followed by the AL and then the L treatment (Fig. 5). The total P (significantly different; *H*_(3,16)_ = 7.82; *p* = 0.0499) was the highest in the A treatment followed by the L and AL treatments (Fig. 5). The control cultures showed relatively, as compared to other treatments in this study, low levels of nitrate, ammonium, and phosphorus and relatively high COD.

**Table 2:** Results for the chemical parameters (nitrate, ammonium, and total phosphorus concentration as well as chemical oxygen demand (COD)) measured in experiments 1 and 2 for the various treatments

treatment	Experiment 1			Experiment 2			
	nitrate (mg/L)	ammonium (mg/L)	COD (mgO_2_/L)	nitrate (mg/L)	ammonium (mg/L)	COD (mgO_2_/L)	total P (mg/L)
C	1.70 ± 0.29	6.1 ± 3.5	167 ± 75	0.19 ± 0.17	0.12 ± 0.06	157 ± 66	1.98 ± 0.76
L	1.65 ± 0.57	11.0 ± 1.7	91 ± 14	0.11 ± 0.00	1.28 ± 0.60	149 ± 16	2.04 ± 0.13
A	1.88 ± 0.15	4.8 ± 1.0	73 ± 5	0.40 ± 0.10	0.05 ± 0.00	48 ± 12	2.74 ± 0.50
AL	1.68 ± 0.41	12.2 ± 1.5	95 ± 15	0.11 ± 0.00	1.21 ± 0.22	119 ± 5.0	1.79 ± 0.09

Mean ± SD

*A* purely *Aspidisca* culture, *L* purely *L. inermis* culture, *AL Aspidisca* and *L. inermis* culture, *C* control

## Discussion

This study provides evidence that *Aspidisca* sp., one of the most frequently observed ciliates in natural and semi-natural macroaggregate systems, can significantly affect the size and form of bacterial aggregates and the rates of mineralization and nitrification. It also demonstrates that *A. cicada* facilitates the population growth of the rotifer *Lecane inermis*, another bacterivorous species of great importance to the functioning of activated sludge [27]. Because the positive effects on bacteria and rotifer growth are associated with changes in the floc physical state, *A. cicada* is a good example of an environmental engineer according to the definition of Jones et al. [50]. If we accept the distinction between ecosystem engineers and keystone species provided by Wright and Jones [51, and citations therein], which states that the former is process-focused while the latter is outcome-focused, it is tempting to claim that *A. cicada* is a keystone species in macroaggregates.

The impact of *A. cicada* on floc characteristics, especially floc size (Table 1, Fig. 3), rotifer proliferation (Fig. 1), and biological activity, here reflected in chemical processes (Table 2), is substantial in both batch cultures (experiment 1) and semi-continuous cultures (experiment 2), and even more importantly, this influence is observable long after its disappearance from the system. The comparison of experiments 1 and 2 shows the condition-independent repeatability of the results, which signifies the strength and consistency of the processes we examined. The additional study on the *A. cicada*-rotifer relationship at a small scale, presented as experiment 3 (Fig. 4), was limited by the *A. cicada* lab culture, which prevented the establishment of treatment replicates. Nevertheless, it validated the important role of the presence of *A. cicada* on rotifer proliferation (the slowest rotifer growth being in the treatment with no *A. cicada*) and revealed some details regarding the intimate rivalry between these two organisms; the initial population growth of rotifers seemed to be the highest in the treatment with the smallest number of *A. cicada* (initial *A. cicada*/rotifer ratio of 1.6 compared to 3.4 and 4.6 in other treatments; Fig. 4b). We offer some plausible hypotheses to explain the actual mechanisms behind the observed interactions.

### Effect of *A. cicada* on flocs

Several authors have discussed the possible effects of ciliated protozoans on the flocculation process in activated sludge [52]. The contribution to flocculation was usually associated with the excretion of various polymeric substances by protozoans, including exploded tricho- and mucocysts, and the production of resting cysts covered with mucus layers [53, 54]. Additionally, the filter-feeding activity of attached and crawling ciliates grazing on suspended bacteria and the smallest aggregates brings many particles to the floc surface, possibly contributing to flocculation [55]. Finally, grazing on freely suspended (not flocculated) bacteria is certainly a selection factor in favor of those bacteria that are able to aggregate. It is not clear to what extent *Aspidisca* ciliates can use suspended bacteria, but it is largely believed that they are specialized consumers of bacteria that are attached to surfaces [38]. Their very unique mouth apparatus with its highly reduced adoral zone of membranelles seems to brush surfaces, collecting loosely attached bacteria [41].

Although the production of EPS seems to be crucial for bacterial and algal flocculation [16], both the formation of aggregates and increased mucus production are also among the defense mechanisms some bacteria and microalgae use against their predators [56]. It has been shown that in the presence of bacterivores, some bacteria that otherwise live as single cells produce filaments or aggregates. The size of these aggregates and larger amounts of EPS appear to be very efficient defenses against some small bacterivorous species. Liu and Buskey [57] observed a significantly reduced grazing rate of *Aspidisca* sp. on cells of the brown tide algae *Aureoumbra lagunensis* when more EPS was secreted by the algae. Fiałkowska and Pajdak-Stós [58] demonstrated that a mat-forming filamentous cyanobacteria, *Phormidium* sp., reduced dispersion and produced more EPS in the presence of a specialized cyanobacteria-consuming ciliate, *Furgasonia blochmanni*. In spite of the continuous presence of cyanobacteria, the ciliates starved and encysted during subsequent days. Yang et al. [59] observed that the cyanobacterium *Microcystis aeruginosa* was induced to form colonies in the presence of the flagellate *Ochromonas* sp.; the colonial form acted as an effective defense against grazing by the flagellate. Further research showed that cells in colonies formed by *M. aeruginosa* subjected to the pressure of *Ochromonas* sp. start to produce more EPS, thanks to which their resistance to grazing increase [60].

In light of these examples, it seems probable that a possible mechanism responsible for the formation of larger flocs in our experiment was a defense response of some bacteria, producing more EPS in the presence of *A. cicada*, which in turn increased the tendency of the smaller aggregates to stick together; EPS act as glue in aggregates, creating larger flocs from smaller ones [61–63]. Another reason of EPS production, the excess of carbon resulting from the shortage of biogens such as nitrogen and phosphorus, may be ignored because, as we have also shown, *Aspidisca* regenerates biogens. The unique characteristics of *A. cicada* together with its tendency to use flocculating bacteria as a food source [38] make it a very specialized scavenger of bacteria that is able to modulate the abundance and size of nitrifying bacteria [26] and in this way promotes the removal of ammonia [26 and this study].

According to our results, *A. cicada* improved the nitrification (nitrate and ammonium) in the experimental cultures. The highest level of phosphorus occurring in the A treatment may signify high mineralization and/or the *A. cicada* feeding preference toward polyphosphate-accumulating bacteria (PAOs); however, this are only speculations.

Our results are consistent with the results of experiment conducted by Grossart and Ploug [64] with the usage of microelectrodes. They drew conclusion that carbon and nitrogen turnover on aggregates resulted in a rapid transformation of particulate organic matter through bacterial growth and grazing.

### Effects of *A. cicada* on *L. inermis*

According to Zimmermann-Timm [5 and citations therein], metazoans do not colonize aggregates smaller than 2 mm. *L. inermis* is not a planktonic rotifer and needs the support of substrate to reproduce. Our previous experience indicates that large numbers of this rotifer cannot be obtained unless culture vessels with an extensive support surface are used. Therefore, the substantially larger abundance of *L. inermis* in the presence of *A. cicada* may be a direct consequence of larger habitat space due to the higher number of larger aggregates in the *Aspidisca*-*Lecane* treatment.

Another possible reason for the observed enhanced growth rate of the rotifer population is higher bacterial production in the presence of *A. cicada*. The higher activity of heterotrophic bacteria can be deduced from significantly lower values of chemical oxygen demand (COD) measured in the supernatant after the sedimentation of bacterial flocs. The higher rate of bacterial decomposition in the presence of bacterivores has already been well documented [65]. Although grazing protozoans usually reduce bacterial abundance, the activity of the remaining bacteria may be much higher compared to the treatments without protozoans. This effect is particularly important for organic substrates that are rich in structural polymers such as cellulose, lignin, or chitin. The decomposition rate of such substrates is limited by the availability of mineral nitrogen and phosphorous. These elements become locked in bacterial cells in the absence of bacterivores. The protozoan grazers release these elements back into the environment, making them available to the remaining bacteria.

Higher bacterial activity in the presence of bacterivores has also been associated with the fact that some of the organic substances released by protozoans are most likely used by bacteria as growth factors. Several studies have demonstrated that bacterivorous protists excrete various compounds like vitamins, co-enzymes or their precursors, amino acids, and nucleotides, which stimulate bacterial growth or activity [66, 67]. Finally, increased bacterial production has also been associated with the locomotory activity of protozoans. The movements of protozoans and their ciliary structures in the direct vicinity of bacterial colonies may be an important factor for reducing the diffusion limitations experienced by bacteria in the viscous world at the microscopic scale [68].

### *Aspidisca-Lecane* coexistence

**Fig. 6:**
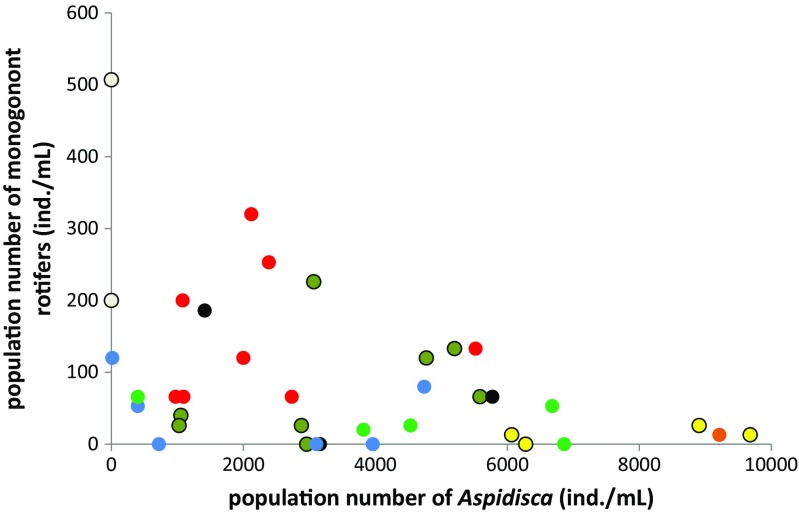
The ratio of monogonont rotifers to ciliates *Aspidisca* sp. estimated for several wastewater treatment plant samples taken at different time points (samples from the same WWTP are *marked in one color*)

In all experiments, *A. cicada* vanished from the system, even in monocultures, which is in accordance with other laboratory studies in which the *A. cicada* stock culture had to be repeatedly inoculated to achieve a required concentration [38, 69]. It therefore seems that this species is very sensitive in laboratory conditions. This is validated by the very low number of reports on laboratory experiments involving this genus [38, 43, 69]. While confronted with rotifers, *A. cicada* always retreated, which could suggest its lower competitive abilities in the face of rotifers. The possibility of *L. inermis* feeding on *A. cicada* may be excluded [70]. However, this laboratory outcome differs considerably from the relationship commonly observed in WWTPs in which *A. cicada* and monogonont rotifers seem to coexist. In the activated sludge habitat, *Aspidisca* sp. is on average one order of magnitude more abundant than rotifers (Fig. 6, M. Sobczyk unpublished data). Related to this discrepancy is that while it is easier to work on small laboratory systems, naturally occurring strong and important relationships may be overlooked and underestimated. One of the reasons why the *A. cicada* populations in our artificial system always tended to decline could be the narrow size spectrum of its prey. According to Luxmy et al. [37], the optimum size of *Aspidisca* sp. prey ranges between 1.5 and 3.3 μm. In our experiments, the “nutrition powder” was inoculated with *Enterobacter* sp. and the bacterial community was additionally enriched by a mixture of bacteria originating from the *A. cicada* culture. The strong initial pressure of *A. cicada* likely could have led to the selection of unavailable bacterial strains or to the triggering of an “escape reaction” in the form of a denser aggregation or the overproduction of protective EPS, as mentioned above. In an activated sludge system that is rich in organisms of different feeding strategies, bacterial morphology is much more diverse. Together with a constant inflow of organic matter (a food source for bacteria), this causes the grazing pressure to be compensated for by a higher bacterial growth rate rather than a defensive strategy.

### Doozers and Fraggles Analogy

To popularize the coexistence of *A. cicada* and *L. inermis* in the macroaggregate system, they may be compared to two races from the *Fraggle Rock* television series by Jim Henson: Doozers and Fraggles. Both are stuck in an isolated micro-world, and the Doozers are the hardworking architects making the sophisticated, openwork constructions that are the favorite snacks of the carefree Fraggles. This fact does not worry the Doozers because without the Fraggles eating their constructions, the Doozers would run out of building space. The presence of *A. cicada* leads to the formation of large, dense flocs that provide additional surface area for *L. inermis* to graze and deposit eggs. Our observations suggest that *L. inermis* is also able to consume the EPS surrounding bacterial colonies [71]. In this way, they damage the possible defense mechanism of bacteria, making them available to ciliates. Moreover, the disintegration of very large flocs by *L. inermis* improves the diffusion of substances necessary for the growth of bacteria hidden in deeper layers. The results of experiment 3 and the data collected from real-scale treatment plants suggest that *L. inermis* and *A. cicada* are able to coexist in a dynamic balance, although the simpler laboratory setup gives rotifers a superior position in competition with *A. cicada*. The coexistence of these two species in activated sludge apparently favors efficient WWTP performance. The presence of *A. cicada* promotes flocculation and ammonia removal, whereas rotifers prevent the selection of bacteria in forms unavailable to ciliates, such as those in very large flocs or filamentous bacteria. Several studies have demonstrated that grazing protozoans can significantly affect biofilm structure by changing its thickness, heterogeneity, porosity, and surface area/volume ratio [19, 72, 73]. Taking into account the general similarity of the EPS matrix in microbial flocs and biofilms [22, 74], there is no reason to believe that similar engineering effects are restricted to biofilms and do not occur in flocs.

It is worth mentioning that we did not avoid contamination by other organisms (other ciliates, flagellates, amoebae) in our experimental cultures, deriving either from the *A. cicada* or the *L. inermis* lab cultures, but this situation seemed to work in favor of our results; even in the presence of contamination, especially in experiment 1, the effect of *A. cicada* on any trait we investigated did not diminish. Additionally, comparisons with the control treatment (Table 2, Figs. 3 and 5) show that the monocultures of other floc organisms must have been less efficient in floc engineering than *A. cicada*.

Most of our knowledge about the possible effects of protozoa on bacteria is based on experiments involving only one or very few identified strains of bacteria and usually only a single protozoan predator [56, 75, 76]. Although these papers clearly show what type of relationships are possible among bacteria and their consumers, we still do not know to what extent such mechanisms affect natural or large-scale engineered systems. A quote from Van Loosdrecht and Henze [77] remains basically still valid: “Possibly, Protozoa form one of the most neglected aspects of the activated sludge process. They are always clearly visible in the microscope, seldom visible in the literature, and never explicitly visible in the models.” This idea can certainly be extended to “aquatic snow” particles in general. Most of the studies concerning the role of protozoans in the activated sludge process have addressed their direct effects related to their grazing on bacteria, mineralization of organic matter, or excretion of EPS [37, 53, 54, 77, 78]. However, we may expect that in complex multispecies floc communities with several trophic levels, indirect effects may also be important. Although such effects are difficult to predict from what is known about the species involved, they may have important community-level consequences. Several examples of such indirect effects between bacteria mediated by a common predator have been described [21, 75, 76].

The most important result of this study is the demonstration of a strong indirect effect of *A. cicada* on the development of an organism from the same level in the food web, the rotifer *Lecane inermis*. This study demonstrates that the actual role of higher trophic-level organisms in activated sludge, and possibly in natural bacterial aggregates in general, may greatly exceed the direct consequences of grazing on bacteria. It also shows that activated sludge is a promising research area for community ecologists.

## Figures and Tables

**Table 4:**
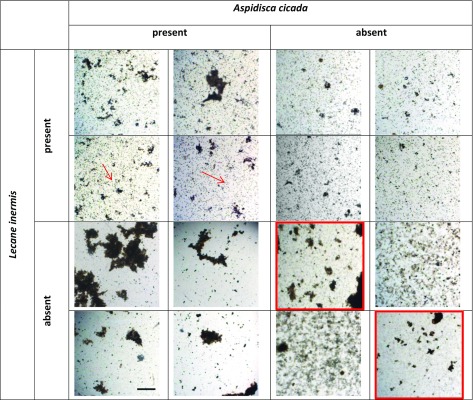
Visual comparison of flock structure in all four replicates in treatments and control in experiment 2, from the subsamples taken on the same date (5 June) and fixed with Lugol solution. Each image taken under a stereomicroscope under the same magnification represents one replicate (1 ml subsample taken from Erlenmeyer flask). Images show the central part of field of view of whole subsamples to avoid vignetting. The largest flocks are visible in A (*Aspidisca*) treatment. *Lecane inermis* individuals are visible as small dark points in AL (*Aspidisca* + *Lecane*) and L (*Lecane*) treatments (red arrows indicate examples). Compact flocks in Control (without *Aspidisca* or *Lecane*) are visible in subsamples contaminated with naked and testate amoebae (images with red edges). Scale bar represents 3 mm

## Appendix

**Table 3:** The mineral contents of spring water (Żywiec, Poland) used as a medium in our experiments

Sum of minerals	230.00 mg/l
Bicarbonate	131.06 mg/l
Fluoride	0.07 mg/l
Magnesium	5.62 mg/l
Calcium	41.69 mg/l
Sodium	9.65 mg/l
